# Structural heat adaptation and education in a rural setting of Pakistan: protocol for a randomised controlled trial

**DOI:** 10.1136/bmjopen-2026-121448

**Published:** 2026-08-31

**Authors:** Jai K Das, Syeda Kanza Naqvi, Ana Bonell, Christopher Burman, Joseph Augustin, Shahmeer Qamar, Arjumand Rizvi, Imran Ahmed Chauhadry, Anjum Abbas Naqvi, Muhammad Khan Jamali, Hassan Naqvi, Farhana Tabassum, Simon Cousens, Zulfiqar A Bhutta

**Affiliations:** 1 Institute for Global Health and Development, Aga Khan University, Karachi, Pakistan; 2 Department of Paediatrics and Child Health, Aga Khan University, Karachi, Pakistan; 3 Medical Research Council Unit the Gambia at London School of Hygiene and Tropical Medicine, Banjul, Gambia; 4 London School of Hygiene & Tropical Medicine, London, UK; 5 The Bartlett School of Architecture, University College London, London, UK; 6 Centre of Excellence in Women and Child Health, The Aga Khan University, Karachi, Pakistan; 7 Centre for Global Child Health, Hospital for Sick Children, Toronto, Ontario, Canada

**Keywords:** Climate Change, Community-Based Participatory Research, Primary Prevention, PUBLIC HEALTH, Quality of Life

## Abstract

**Introduction:**

Pakistan is one of the countries most affected by climate change, where peak temperatures in certain areas frequently exceed 40°C. Despite evidence that vernacular designs and behavioural adjustments can reduce indoor temperatures, heat‐adaptation efforts remain concentrated in urban or high‐resource settings, with limited evidence for vulnerable rural populations in low- and middle-income countries, including Pakistan.

**Methods and analysis:**

This trial aims to evaluate the effectiveness of a comprehensive Resilience and Heat Adaptation Bundle (ReHAB) that integrates behavioural and structural interventions to reduce heat-related morbidity and mortality and improve internal ambient conditions in rural settings of Pakistan. This study is a prospective, cluster-randomised trial, comprising 22 clusters allocated to intervention and control arms in a 1:1 ratio. The ReHAB intervention combines community education and awareness with targeted structural improvements, including roof and wall treatments, enhanced shading, improved ventilation, sustainable energy installations and communal outdoor shelters. Health and behavioural data will be collected through household surveys, while wearable devices and environmental sensors will measure individual heat exposure and record indoor and outdoor thermal conditions.

**Ethics and dissemination:**

The study has received ethical approval from the Aga Khan University (AKU) (ref: 2025–11559-35625) and National Bioethics Committee (ref: 4-87/NBCR-1065/23/1736). Written informed consent will be obtained from all participants before enrolment. Referral pathways to healthcare facilities will be established to ensure timely management of complications. Findings will be disseminated through peer-reviewed publications, scientific conferences and engagement with policymakers and public health stakeholders. Results will also be shared with participants and communities through meetings and informal sessions to raise awareness and support evidence-based heat adaptation.

**Trial registration number:**

NCT06983483.

**Strengths and limitations of this study**

- The trial will evaluate heat adaptation strategies involving structural housing modifications (retrofitting) and behavioural interventions in rural communities.
- The use of weather stations, environmental monitoring devices and personal wearable sensors will enable comprehensive measurement of household thermal environments, individual heat exposure, health outcomes and physiological responses.
- Strong community engagement and participatory implementation approaches are incorporated to enhance acceptability, local ownership and long-term sustainability of the interventions.
- Compliance challenges related to wearable devices, including potential discomfort during hot weather and inconsistent usage, may affect completeness of physiological monitoring data.

## Background

 Anthropogenic greenhouse gas emissions have raised global temperatures by approximately 1.1°C since the late 19th century^1^ and as a result, extreme heat events are becoming more frequent, prolonged and intense. Marine heatwaves have tripled in duration since the 1940s,^2^ while land-surface heatwaves have intensified, contributing to rising heat-related morbidity and mortality worldwide,^3^ with nearly 37% of heat-related deaths being attributed to climate change over the past two decades.^4^ A multicountry analysis across 732 locations in 43 countries suggested that mean temperatures had risen by 0.26°C per decade between 2000 and 2019, with heat-related mortality increasing proportionally.^5^ The most vulnerable populations are affected disproportionately; heat-related mortality among older adults increased by approximately 85% between 2000–2004 and 2017–2021.^6^ Extreme heat also reduces the quality of life by disrupting daily functioning, physical comfort, mobility and productivity, with downstream effects on economic stability and psychological well-being.^7^

Pakistan faces the compound challenges of climate change, poverty and infrastructure deficits. The country experiences recurrent heatwaves including the catastrophic 2015 heatwave, which resulted in approximately 700 deaths in Sindh,^8^ and the 2024 heatwave, during which temperatures in rural Sindh approached 50°C for several consecutive days and overwhelmed local health facilities with cases of heatstroke and gastroenteritis.^9^ Rural populations, many of whom are engaged in outdoor manual labour, face additional vulnerabilities as over 50% lack piped water and rely on hand-pumps or canals, while prolonged power outages limit access to fans, refrigeration and other cooling measures.^10 11^ Primary healthcare centres often lack basic facilities, which may delay treatment for heat-related illnesses and increase preventable morbidity and mortality.^12^

Historically, the rural communities adopted passive, low-cost strategies embedded in vernacular architecture, including use of mud, brick and stone construction, wind catchers, thermal mass, strategic building orientation, courtyards and shaded spaces. These approaches help to reduce indoor heat and maintain comfort without reliance on electricity.^13–15^ However, with time these indigenous practices are being replaced by concrete structures that are often poorly oriented for heat protection and ventilation. Improving the housing infrastructure is therefore important, alongside behavioural adaptations such as appropriate work-rest schedules, wearing loose cotton clothing and modifying food and fluid intake.

Although the recent heatwave management plans and early warning systems have improved emergency response, they primarily address acute exposure and do not sufficiently focus on preventive measures and reducing chronic household-level heat stress in resource-constrained rural communities. Most evidence on structural heat adaptation interventions comes from high-resource or urban settings.^16^ A few studies have evaluated simple community-level strategies including a community health worker-led education programme in Karachi that reduced unscheduled hospital visits,^17^ while agroforestry and tree-based interventions in rural low- and middle-income country (LMIC) settings have demonstrated modest health and economic benefits, although their health impacts remain underquantified.^18^

These gaps highlight the need for context-specific, low-cost heat adaptation strategies tailored to the rural socioeconomic and infrastructural realities. The Structural Heat Adaptation and Education in Rural Settings trial will evaluate one of the first heat adaptation and resilience bundles combining vernacular retrofits, community mobilisation and targeted behaviour change in rural context to generate evidence for scalable, community-led climate adaptation in a heat-vulnerable region.

## Methodology

### Objective

#### Primary

1. To assess the feasibility and effectiveness of a Resilience and Heat Adaptation Bundle (ReHAB) on heat-related health risks for vulnerable populations in rural settings of Pakistan.
2. To assess the effectiveness of ReHAB on quality of life of vulnerable populations in rural settings of Pakistan.

#### Secondary

1. To evaluate the effectiveness of ReHAB on indoor heat in rural settings.
2. To evaluate the impact of ReHAB on key physiological variables (sleep quality, heart rate, physical activity and energy expenditure) in adults residing in rural settings.

### Study setting

This study will be conducted in a representative rural district of Matiari which has a total area of 1417 square kilometres and a population of 849 383.^19^ It comprises three subdistrict administrative units (tehsils); Hala, Matiari and Saeedabad. Each tehsil is further divided into multiple union councils. During summer months, the average outdoor temperature usually ranges from 38°C to 42°C. The hottest period extends from May to August, with average daily maximum temperatures surpassing 50°C.^20^ The community residing in this setting belongs to a low socioeconomic background with limited access to basic amenities with agriculture and livestock as their main source of income.

### Study design

**Figure 1:**
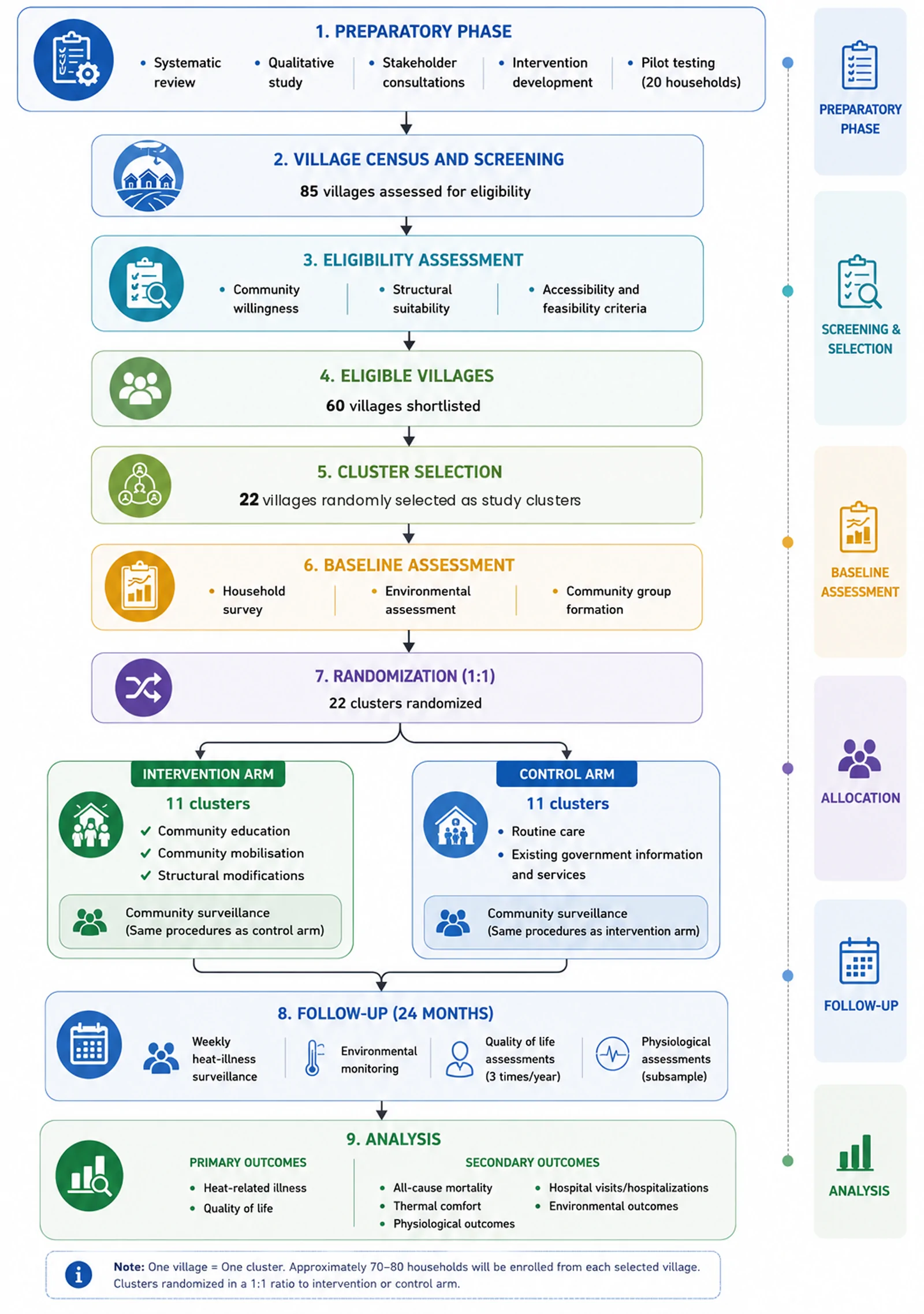
Study design diagram.

This study is a 2-year prospective, cluster-randomised controlled trial designed to evaluate the effectiveness of ReHAB intervention in rural settings. One village will serve as one cluster with 70–80 households randomized, and these clusters will be randomly assigned to either the intervention or control arm in a 1:1 ratio (figure 1). Due to the nature of the intervention, blinding of participants and researchers will not be feasible, but data analysis would be blinded. The trial is registered on clinicaltrial.gov with trial registration number NCT06983483. The study field activities will run from 1 January 2026 to 31 December 2027.

### Population

The population of this trial will include all members of the households within the selected clusters. A subset of eligible adults aged 18–60 years will be randomly selected for detailed physiological and personal heat exposure assessments.

### Exclusion criteria

We will exclude any clusters that present significant accessibility or security concerns, have inadequate structural stability for retrofitting or limited community cooperation. For the subset population, individuals unwilling to wear both (Fitbit and temp-u) devices simultaneously, individuals with cognitive or severe mental health conditions that may impair their ability to provide informed consent or comply with study procedures, and individuals with severe physical disabilities that would limit participation in physiological monitoring will be excluded.

### Outcomes

**Figure 2:**
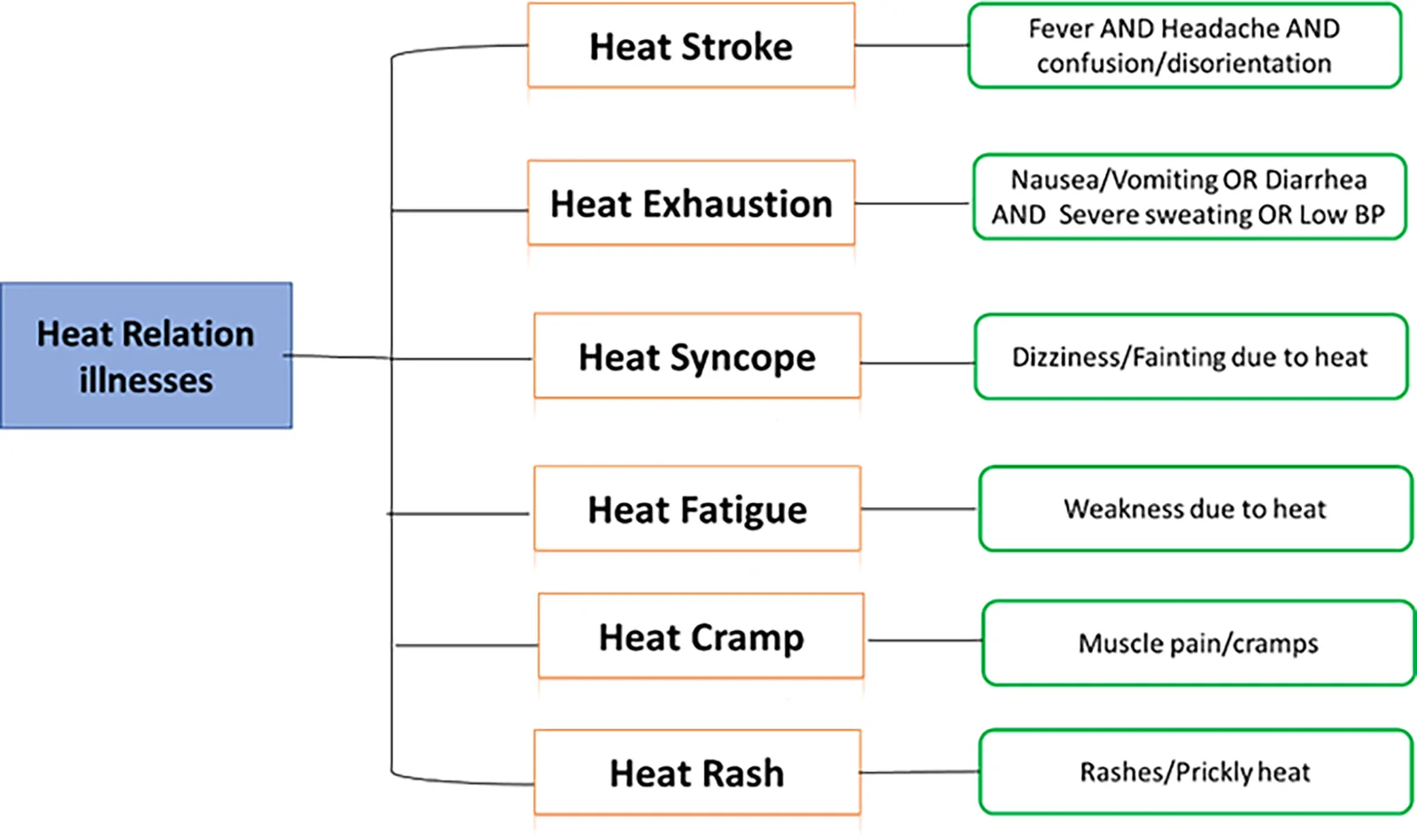
Classification of heat illness symptoms. BP, blood pressure.

All primary, secondary and outcomes assessed for subset of population along with definitions are provided in table 1 and figure 2.

**Table 1:** Outcomes

Primary outcomes	
Heat-related illness	Defined as a documented heat condition/illness according to the International Classification of Disease-11th Revision and institutional classification which classifies heat-related illness as heat stroke, heat syncope, heat exhaustion, heat fatigue, heat cramps and heat rash (figure 2).^30 31^
Quality of life	Quality of life would be assessed according to the WHOQOL-BREF* tool and assessed during two consecutive peak summer and winter seasons.

Secondary outcomes	
Health outcomes	
All-cause mortality	Defined as the total number of deaths in the study population over the study period
Hospital visit/hospitalisation	Defined as the number of hospital admissions (primary, secondary or tertiary facilities) during summers
Environmental outcomes	
Heat Index	Indoor heat index (temperature and humidity)^32^ measured through fixed weather devices
Universal Thermal Climate Index	Defined as a biometeorological index that describes the human body’s physiological response to the environment. It integrates air temperature, humidity, wind speed and radiant temperature to assess the overall thermal stress on the human body
Thermal comfort	Defined as change in the condition of mind that expresses satisfaction with the thermal environment
Outcomes measured for subset of population (individual level)	
Personalised heat exposure	Defined as mean daily external temperature each individual experiences (indoors and outdoors) as recorded by a wearable temperature monitor.
Heart rate	Defined as mean heart rate measurements (beats per minute) taken at 1-hour interval as recorded by wearable android device
Sleep quality	Defined as a biological process that alternates between non-rapid eye movement sleep and rapid eye movement sleep as recorded through a wearable android device
Physical activity	Defined as mean daily duration of sedentary, lightly active, moderately active and very active as measured using a wearable android device.
Energy expenditure	Defined as average total number of calories burned in a day recorded using a wearable android device.
Economic outcomes	
Cost-effectiveness	Measured as the difference in cost divided by the difference in health effect between the intervention and control groups

* WHO Quality of Life Brief tool.

### Sample size

Sample sizes were calculated for both the primary outcomes to provide at least 80% power at the 5% significance level. Based on heat-related symptoms and illness prevalence of 28.9%^21^ and an estimated intracluster correlation coefficient (ICC) of 0.0122, we calculate that 11 clusters per arm (22 clusters in total) with 70 to 80 households per cluster are needed to detect a 30% relative difference in heat-related illnesses while allowing for a 10% attrition rate. We treat 28.9% as a conservative estimate given the longitudinal nature of the primary outcome, as cumulative prevalence over the surveillance period is expected to exceed this single-time point prevalence. To assess quality of life changes, we estimate that with 11 clusters per arm 60 households per cluster are required based on the variability in baseline WHO Quality of Life Brief tool (WHOQOL-BREF) scores reported in Lodhi *et al*,^22^ which documented an average score of 68 (18.0) to detect a difference of 4 points in quality of life score assuming an ICC of 0.015. As the heat-related illness outcome required the larger sample size, the final enrolment target was set at 70–80 households per cluster, which also provides adequate power for the quality-of-life outcome. To assess physiological heat strain, we calculated that a subsample of eight adults (aged 18 years to 60 years) per cluster (balanced by gender) would be required to detect a 15% change in average heart rate, among those who are exposed to heat stress assuming a baseline of 66±12 bpm,^23^ an ICC of 0.1736 and 90% power. Given the complexity of the assessment process, we anticipate a higher attrition rate in this subset and have inflated the sample size by 30% to 10 individuals.

For binary outcomes, the ICC was derived from an assumed cluster-level prevalence variation of ±10%, with the coefficient of variation (CV) estimated from this range and the ICC subsequently calculated using the relationship ICC=k²×P/(1−P), where k is the assumed between-cluster CV of the prevalence, following Pagel *et al*.^24^ For continuous outcomes, the ICC was estimated as the ratio of between-cluster variance to total variance,^25^ with total variance sourced from reference studies and a minimum between-cluster difference of ±9 units assumed. Each co-primary outcome is independently powered at 80% or above. To control the family-wise type I error rate across the two co-primary outcomes, hypotheses will be tested using a prespecified fixed-sequence (hierarchical) procedure: heat-related illness will be tested first at the two-sided 0.05 level, and quality of life will be tested at 0.05 only if the first test is significant. As each outcome is independently powered, this procedure incurs no loss of power. Quality of life is assessed at three time points per year over 2 years; the cross-sectional calculation above is therefore conservative, as the planned repeated-measures mixed-effects analysis increases precision by combining information across visits.

### Formative phase

The ReHAB intervention comprises two principal components: (1) community education and awareness and (2) structural modifications for heat adaptation (figure 1). The formative phase consisted of multiple activities designed to determine the specific intervention components which would be feasible, acceptable and cost-effective.

### Systematic review

A systematic review of community-based heat adaptation interventions in LMICs was undertaken.^26^ A total of 185 studies were reviewed to identify effective approaches and to highlight existing gaps in adaptation strategies. Findings from this review informed the development of a list of evidence-based interventions, which were subsequently used to guide discussions with communities, stakeholders and policymakers.

### Qualitative study

The second component of the formative phase involved in-depth interviews and focus group discussions with key stakeholders. These included policymakers, government officials across national, provincial and local levels, civil society representatives and community members, with particular emphasis on women. Insights from this work were used to assess potential educational and structural interventions, as well as appropriate delivery mechanisms. Detailed findings will be reported and published separately.

### Consultative workshops

A series of consultative workshops were conducted with a broad range of stakeholders, including municipal authorities, relevant government departments, technical experts and community representatives. These engagements were used to refine and finalise the intervention design.

### Pilot study

A pilot study was conducted in a single non-study village involving 20 households to assess participant recruitment, acceptability and feasibility of intervention, adherence to wearable devices and completeness of data collection procedures. The pilot was conducted from 10 April 2025 to 15 June 2025. During this phase, the complete set of intervention components were implemented alongside community engagement activities. Environmental data were collected to evaluate the effects on indoor and outdoor temperatures. Following completion, the findings were analysed and reviewed in collaboration with stakeholders and subject-matter experts to inform further refinement of the intervention.

### Intervention

#### Intervention arm: ReHAB

The ReHAB intervention was developed based on findings from the formative phase and comprises two components: (1) community education and awareness and (2) participatory structural modifications to support local heat adaptation. Behavioural interventions will be delivered through community mobilisation, while structural interventions will focus on modifying existing structures to reduce indoor temperatures. As the unit of randomisation is the cluster, so intervention delivery is standardised at the cluster level, with all intervention clusters receiving the same community education and mobilisation package and a proportional distribution of structural interventions.

##### Community education and awareness

The educational component of ReHAB was developed based on findings from the formative phase, including systematic review, qualitative work, stakeholder consultations and pilot testing. Community mobilisation will be implemented throughout the intervention. Community groups (CGs) will be established across all 11 intervention clusters, with separate male and female representation. Each CG (6–8 members) will include local government representatives, elders, religious leaders and male and female community members. CGs will lead mobilisation activities and support surveillance of the primary outcome (heat illness).

Activities will include dissemination of heatwave warnings, awareness sessions and door-to-door outreach on heat-health practices (hydration, clothing and activity scheduling). CGs will maintain logbooks to record healthcare visits within their catchment areas and attendance in community meetings. Practical demonstrations of cooling strategies (eg, shade setup, light clothing, wet towels, misting and work–rest–shade cycles) will be conducted, supported by Information, Education and Communication (IEC) materials. This structured, behaviour-focused delivery aims to ensure both the uptake and sustainability of protective cooling practices. Community hydration measures (eg, temporary drinking water facilities) will also be implemented to reduce dehydration among outdoor workers.

###### IEC materials

IEC materials will be developed based on formative findings and include:

- Posters.
- Pictorial brochures/flip charts.
- Short video messages in the local languages (Sindhi, Pashto and Urdu).

Materials will be distributed during CG meetings and follow-ups, while videos will be shared via WhatsApp using disappearing message settings to minimise re-distribution and possible contamination.

###### Training

CGs will be trained on heat prevention messaging, use of temperature monitoring devices and data loggers, and community-based surveillance of heat-related illness. They will also be trained to communicate structural intervention decisions effectively.

###### Sessions

Sessions will be led by CGs and conducted separately for men and women, including initial triggering meetings and follow-up sessions supervised by research staff.

- Summer (April to June):
  - Fortnightly community meetings.
  - Weekly WhatsApp video messages.
  - Weekly weather alerts.
- Late summer (July to October)
  - Monthly community meetings.
  - Weekly WhatsApp video messages.
  - Weekly weather alerts.
- Winter (November to March):
  - Community meetings every other month.
  - No WhatsApp video messages.

### Structural modification

**Figure 3:**
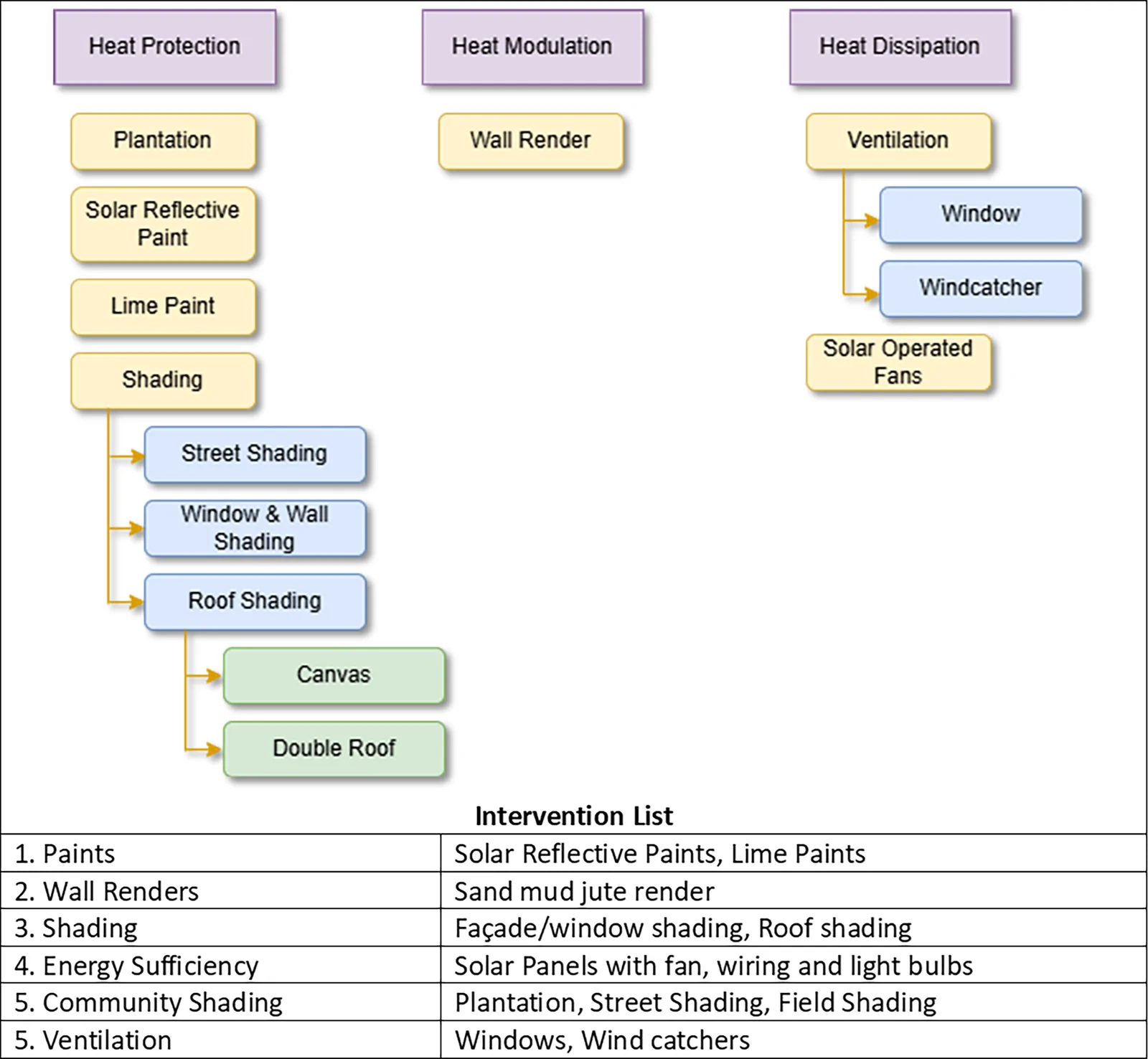
Categorisation of structural interventions.

**Figure 4:**
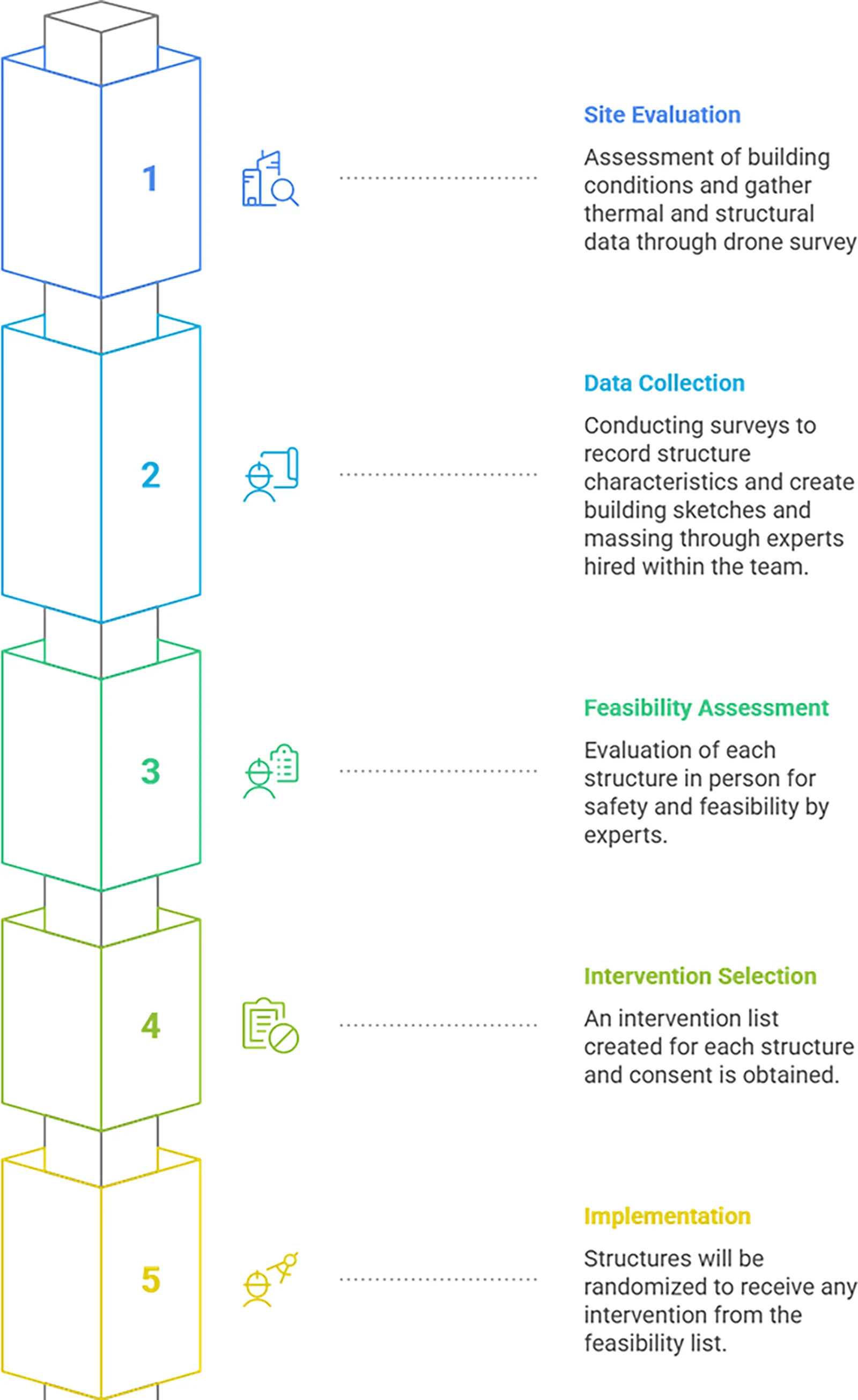
Building survey and intervention process.

This component will include modifications to existing structures. The structural interventions considered for each household and the communal interventions will comprise the possible options as described in figure 3. The household level interventions are targeted to reduce indoor temperature, and the community interventions will play a role in reducing the effects of high heat for outdoor workers and the community as a whole. The interventions are divided into three categories, heat protection, heat modulation and heat dissipation^27^ (figure 3). Each suggested intervention will be evaluated on site and homeowners will be informed and their consent will be obtained. All interventions will be delivered proportionately in each cluster, that is, equal number of each intervention type in every cluster. Interventions will be decided based on building materials, strength and efficiency, overshadowing, ventilation conditions and consent of residents. Apart from this, all interventions will be assessed for safety in terms of structural safety and resident safety (figure 4). Structural modifications will be documented through building assessments and implementation records and experts, architects and structural engineers will be hired to maintain real time monitoring and evaluation.

These interventions will be delivered based on a community participatory approach where the community and project will share the cost of intervention to improve ownership as shown in a previous trial.^28^ The community contribution will be at least 30% of the total cost at cluster level and this contribution can be monetary or non-monetary, for example, provision of labour or supplies and individual households will not be excluded solely because of inability to contribute financially.

### Control arm

The control arm will receive routine care and usual public-sector health services and general government or media heat-related information which is available to the wider population with no additional intervention. Selected community representatives will conduct surveillance for heat-related illnesses.

### Data collection

Data collectors will undergo 1 week of training on data collection tools, protocols, device handling and procedures. Competency will be assessed through field-based practice questionnaires.

### Line listing

A household census will be conducted across 85 villages in Matiari to assess eligibility and willingness of the community. Data collected will include household ID, number of stories, household size, presence of elderly, children and women of reproductive age, duration of residence and primary floor and roof materials. Villages will then be reassessed for feasibility, including community cooperation and structural suitability for retrofitting. Based on this, 60 clusters will be shortlisted and 22 clusters will be randomly selected and allocated in a 1:1 ratio to the intervention and control arms.

### Cluster randomisation

We will employ a constrained randomisation procedure as described by Morgan and Rubin,^29^ rather than relying on a single random allocation, the randomisation process will be implemented using the Stata ‘randomise’ command, which generates multiple allocation schemes in the background and evaluates balance across prespecified baseline covariates. An allocation achieving an acceptable level of balance between study arms at the cluster level will then be selected for the trial.

Clusters will be randomly assigned by an independent statistician to the intervention (ReHAB) or control arm. Allocations will be assessed for balance on population density, structural type (single vs double story) and roof type (permanent, semipermanent, natural). To minimise contamination, only non-adjacent villages will be selected as clusters, with a minimum separation distance of approximately 1–2 km.

For in-depth assessments, eight households per arm will be selected using simple random sampling and one adult will be selected from each household using the Kish grid. If the selected individual refuses, another eligible adult will be approached; if all adults of a particular household refuse, the household will be replaced with the next in sequence. Male and female participants will be selected systematically.

### Household data collection

Baseline, periodic (monthly/weekly) and endline surveys (Table 3) will be conducted in all the 22 intervention and control clusters. Data will be collected on demographics (household size, socioeconomic status, education, occupation, ethnicity), healthcare-seeking behaviour, mental health (WHO well-being scale), food security, dietary diversity, comorbidities, medications and symptoms of heat-related illness (box 1).

Box 1 **Section for data collection tool**

**Description of modules and their sections**

Module-A: Household identification and demographic information

Section-A1: Household identification and consent

Section-A2: Household members’ information

Section-A3: Socioeconomic status of household

Section-A4: Building survey

Section-A5: Household water and sanitation status

Module-B: Household food insecurity, dietary diversity and source assessment

Section-B1: Household Food Insecurity Experience Scale

Section-B2: Household Dietary Diversity and Source Assessment

Module-C: Health and hygiene assessment of adult participants

Section-C1: General health and disabilities

Section-C2: Mental health

Section-C3: Hygiene practices (hand washing, dental hygiene, bath (menstrual hygiene for females))

Module-D: Heat exposure/sleep/dietary assessment

Section D1: Work and sun exposure

Section D2: At-home heat exposure

Section D3: Heat stress experiences, food security and adaptation strategies

Section D4: Knowledge about heat-related health problems, existing heat adaptation and prevention strategies

Section-D5: Sleep

Section-D6: Quality of life

The questionnaire was adapted from validated tools used in similar settings, with modifications for local relevance. It was translated into the local language and back translated into English to ensure accuracy.

Data on signs and symptoms of heat-related illness will be collected through multiple sources to minimise missed events during the study period in the intervention and control clusters. Suspected heat-related illness episodes identified through community surveillance, household surveys or health facility records will be classified according to the predefined symptom-based algorithm by study data collectors as presented in figure 2 and categorised using International Classification of Disease, 11th Revision and institutional criteria. Events that would be identified through community surveillance will be visited by data collectors who will use the same survey tool, and reported health facility visits will be verified through facility records, where feasible. Quality of life data will be obtained through repeated household surveys, with the selected index adult serving as the respondent (Table 3).

Quantitative data will be collected using handheld devices with a custom-built Java application, with MySQL and SQLite used for data storage. Built-in range checks, consistency checks and skip patterns will minimise data entry errors. Any discrepancies will be verified and resolved through follow-up with data collectors. Each participant will be assigned a unique identifier generated by the purpose-built Android application to maintain anonymity. All data will be securely stored behind firewalls and fully anonymised prior to analysis.

In the second year of intervention, 5 mL venous blood samples will be collected from participants in both intervention and control households during peak heat time. Samples will be obtained by trained phlebotomists using standard aseptic techniques and stored in a biobank according to established protocols. These samples may be used for future exploratory analysis of heat-related biomarkers which have not been specified yet.

### Environmental data collection

Environmental assessment will include thermal drone surveys to evaluate heat accumulation and dissipation across structures. Surveys will be conducted at baseline and annually at three time points: sunrise (within 2 hours), mid-day (12:00–16:00) and sunset (1 hour before and after sunset).

Indoor temperatures will be recorded in all households using thermochron ibuttons at 20-minute intervals. Outdoor temperature and humidity will be measured using HOBO weather stations and Kestrel Drop 2 devices (also at 20-minute intervals). These devices have been validated globally and serve as standard environmental monitoring tools. In addition, local weather station data will be used to obtain daily measures of ambient temperature, relative humidity, wind speed and solar radiation throughout the study period. Heat stress indices will be derived from these parameters, including the Universal Thermal Climate Index (UTCI) and Wet Bulb Globe Temperature.

Designated staff will routinely retrieve data from installed and wearable devices and return them after transfer. In areas with limited internet connectivity, data will be manually exported onto secure USB devices and stored on password-protected laptops. All data will be archived in a secure repository at Aga Khan University (AKU), Karachi, with access restricted to authorised personnel via institutional network credentials. Role-based permissions will ensure confidentiality. The repository will be synchronised daily with a secure remote backup to prevent data loss or corruption.

### Devices

The devices and their utilisation for outcome measurements are provided in table 2.

**Table 2:** Devices used for outcome measures

Outcome measures	Device	Placement
Individual and household level exposure	Thermochron ibuttons, Temp-U which record temperature and humidity	Put at select households in the intervention and control clusters within the main living room, midway between floor and ceiling and away from any direct heat source. Devices will be given to individuals selected for subgroup for individual exposure assessment
Environmental exposure	Kestrel 5400 which will record multiple environmental parameters like wet bulb globe temperature, solar radiation, barometric pressure, wind speed and humidity	One device rotated in each cluster
Individual physiological outcomes	Fitbit Inspire 3 wearable fitness tracker that will track heart rate, sleep cycles, activity levels and energy consumption	Given to the subset population selected in the intervention and control clusters
Weather	Kestrel drop D2 and HOBO U-30 weather stations	One weather station placed at the field office as a central location and one Kestrel drop D2 placed in each cluster

### Data collection from subset population

Monthly data will be collected from the subset population to provide detailed insights into heat stress and intervention effectiveness (table 3). This will include assessment of thermal comfort, mental health and heat exposure.

**Table 3:** Schedule of outcome assessments and follow-up activities

Parameter	Time points	Details	Devices/tools	Population/unit
Primary outcomes				
All heat-related illness	Weekly during peak summers	Heat-related illnesses	Weekly community survey, health facility data, community surveillance	All members of included households
Quality of life	Mid of April, July and December for two consecutive years	Quality of life mean score	WHOQOL-BREF* questionnaire	Indexed adults of all included households
Secondary outcomes				
All-cause mortality	Weekly during peak summers	Any mortality occurring within the included clusters	Weekly community survey, health facility data, community surveillance	All members of included households
Hospital visit/hospitalisation	Weekly during peak summers	Any hospital admission or hospital visit occurring within the included clusters	Weekly community survey, health facility data, community surveillance	All members of included households
Indoor Heat Index	Continuous throughout the year	Indoor data loggers	Ibutton, Kestrel Drop D2, Temp-U	All included households
Thermal comfort (home)	Monthly	Self-reported comfort levels	Thermal comfort survey adapted from CHEQ-5‡	Indexed adults randomised for subgroup
UTCI	Continuous throughout summers	Outdoor only	Kestrel Drop D2, Kestrel 5400, HOBO weather station	Overall catchment area
Outcomes measured for subset of population				
Personalised heat exposure	Baseline, Monthly	Occupation, sun exposure, illness history, daily routine	Structured survey tool (adapted from HotHaps) and Temp-U	Indexed adults randomised for subgroup
Physiological strain markers	Continuous throughout summers	Heart rate, sleep cycles, VO₂ max†	Fitbit Inspire 3	Indexed adults randomised for subgroup

Participant compliance will be monitored through spot checks and periodic review of device data. For Fitbit devices, heart rate data will be reviewed, as prolonged absence of heart rate recordings may indicate that the device was not worn. Similarly, iButton and Temp-U data will be checked periodically for implausible temperature spikes or sudden drops, which may indicate non-compliance or incorrect device use.

* WHO Quality of Life Brief tool.

† Maximum Volume of Oxygen.

‡ Cold and Heat Evaluation Questionnaire.

UTCI, Universal Thermal Climate Index.

Individual heat exposure will be measured among a subset of participants (eight per cluster) using thermochron ibuttons or TempU devices attached to clothing. Participants will be instructed to always wear the device and to place it beside their bed while sleeping.

Participants in the subset will also be provided with Fitbit Inspire 3 devices to capture physiological parameters, including heart rate, body temperature variation, sleep, energy expenditure and physical activity. These devices will be synchronised weekly with the AKU server, charged and returned to participants.

### Statistical analysis

The trial has two co-primary outcomes, heat-related illness and quality of life. These outcomes will be evaluated independently to assess the effectiveness of the intervention. All statistical analyses will be conducted using STATA V.18.0 (https://www.stata.com), following an intention-to-treat approach while accounting for the cluster-randomised trial design. Baseline characteristics will be analysed to assess comparability between intervention and control arms. Descriptive statistics will summarise household and participant characteristics, and any important imbalances at baseline will be adjusted for in the analysis. No interim analysis or formal stopping guidelines are planned given the low-risk nature of the intervention.

Statistical analyses will account for the cluster-randomised design by incorporating clustering through random effects or robust variance estimators, as appropriate. For outcomes measured repeatedly over time, within-participant correlation will be accommodated using repeated-measures mixed-effects models. Intervention assignment and seasonal variation will be included as fixed effects in all applicable models, while relevant baseline covariates and baseline outcome values (where available) will be adjusted for. Model assumptions, including overdispersion for count outcomes, will be assessed and alternative modelling approaches (eg, negative binomial models) will be used where appropriate. Missing data will be examined and handled using complete-case analyses or multiple imputations, depending on the extent and pattern of missingness. Sensitivity analyses will be performed to assess the robustness of findings to different assumptions regarding missing data.

#### Primary outcome analysis

For each participant, a summary record will be constructed comprising the number of heat-related illness episodes and the number of assessment occasions (or person-time) over which the individual was observed. To assess the intervention effect, a Poisson regression model with cluster-level random intercepts will be used, accounting for intracluster correlation. The number of heat-related illness episodes/events will serve as the numerator, while person-weeks at risk will be used as the denominator. Results will be presented as incidence rate ratios (IRRs) with 95% CIs.

Duplicate reports identified across multiple sources will be flagged in the database using participant ID, household ID, date of onset, symptoms and care-seeking information to ensure that each event is counted only once. Symptoms or events continuing from a previous reporting period will be considered part of the same illness episode. A new episode will be defined as either the onset of symptoms following a symptom-free interval of at least 2 days after the previous episode or the occurrence of additional new symptoms in a participant with ongoing previously reported symptoms. For quality-of-life assessments, changes from year one to year two will be compared across three assessment points per year. Summer and winter quality-of-life scores will also be analysed separately. Results will be reported as adjusted mean differences with 95% CIs.

#### Secondary outcome analysis

Secondary outcomes will be analysed within the same cluster randomised framework, accounting for clustering at the design level using mixed-effects models or generalised estimating equations as appropriate to the distribution of each outcome. All-cause mortality and hospital visits/hospitalisations, measured as counts or rare events, will be analysed using mixed-effects Poisson or logistic models (or Cox proportional hazards models where individual-level time-to-event data are available), with results reported as IRRs, risk ratios or HRs with 95% CIs. Indoor heat index and UTCI, both continuous environmental exposure measures, will be assessed using linear mixed-effects models with random intercepts for clusters and adjustment for temporal variation to estimate mean differences between trial arms. Thermal comfort, measured either as a continuous score or ordinal response, will be analysed using linear mixed models or ordinal logistic mixed-effects models depending on scale properties. Subgroup analyses will be conducted by including interaction terms between intervention assignment and key effect modifiers such as age (Women of reproductive age, children and elderly), sex and occupational exposure. All models will be adjusted for relevant covariates where appropriate, and robustness will be assessed through sensitivity analyses including alternative correlation structures. Cost-effectiveness analysis will be conducted to evaluate the economic feasibility of different adaptation strategies.

Analysis for subset outcomes measured in the subset of the population will be analysed using multilevel mixed-effects models to account for repeated measurements within individuals and clustering at the household or cluster level, as appropriate. Personalised heat exposure, collected longitudinally, will be analysed as a continuous outcome using linear mixed-effects models incorporating time-varying covariates such as occupation, daily activity patterns and reported illness history. Physiological strain markers obtained from wearable devices (eg, heart rate, sleep metrics and related indices) will be analysed using hierarchical mixed-effects models to estimate intervention effects on both average levels and temporal trajectories over the study period, with additional smoothing or functional approaches considered for high-frequency data. All analyses will report adjusted mean differences with corresponding 95% CIs and will explore heterogeneity in effects over time and across participant characteristics where data permit.

### Ethical considerations and dissemination

This study will be conducted in accordance with strict ethical standards to ensure the safety, dignity and rights of all participants. Ethical approval has been obtained from the Ethical Review Committee of Aga Khan University (ref: 2025–11559-35625) and the National Bioethics Committee (ref: 4–87/NBCR-1065/23/1736). Written informed consent will be obtained from all households and community gatekeepers; for participants with limited literacy, oral consent will be documented through signature or thumb impression in the presence of a witness. Participants will be fully informed about study objectives, procedures, potential risks and benefits. Separate consent will be obtained for wearable device monitoring and structural interventions (online supplemental file 1).

An additional consent process will be conducted for blood sample collection, detailing the purpose, volume, analyses, storage duration and potential future use in ethically approved research. Participants will be informed of minimal risks associated with venipuncture, including mild pain, bruising, dizziness or rare infection, and appropriate precautions will be taken. Samples will be processed and stored for at least 7 years in a secure, access-controlled biobank compliant with national standards. All specimens will be de-identified using unique study codes, with personal identifiers stored separately in password-protected databases accessible only to authorised personnel. Participants may withdraw consent for storage and request sample destruction at any time (online supplemental file 2).

Confidentiality will be maintained through anonymisation of all participant data using unique identifiers. Personal information will be stored separately, and data will be collected on encrypted devices and stored on password-protected servers at AKU, with access restricted to authorised personnel. No data will be shared with third parties without consent, and findings will be reported in aggregate form. Any discomfort related to wearable devices or monitoring will be assessed, and participants may withdraw at any stage. Structural and environmental interventions will be implemented in collaboration with communities to minimise risk and burden. Installed household-level materials will remain with participating households, while communal infrastructure will remain under community ownership.

Efforts will be made to ensure equitable inclusion of marginalised groups, including women, older adults and outdoor workers. Community engagement will involve both men and women, with materials provided in Sindhi, Pashto and Urdu. Interventions will be co-developed with communities to enhance acceptability and ownership.

Any ethical concerns or protocol deviations will be reported promptly, and corrective measures will be implemented. The study will adhere to high standards of research integrity while prioritising participant safety and well-being.

Findings will be disseminated through peer-reviewed publications, scientific conferences and engagement with policymakers and public health stakeholders. Results will also be shared with participants and communities through meetings and informal sessions to raise awareness and support evidence-based heat adaptation.

## Supplementary material

10.1136/bmjopen-2026-121448 online supplemental file 1

10.1136/bmjopen-2026-121448 online supplemental file 2
