# Supplementary material for: Structural heat adaptation and education in a rural setting of Pakistan: protocol for a randomised controlled trial
Source: BMJ Open. 2026 Aug 31;16(8):e121448. doi: 10.1136/bmjopen-2026-121448 (PMC13536121; doi:10.1136/bmjopen-2026-121448)
Supplement: Supplementary file 1 [file bmjopen-2026-121448.reviewer_comments.pdf]

PEER REVIEW HISTORY

BMJ Open publishes all reviews undertaken for accepted manuscripts. Reviewers are asked to complete a checklist review form and are provided with free text boxes to elaborate on their assessment. These free text comments are reproduced below.

ARTICLE DETAILS

Title (Provisional)

Protocol for a Randomized Controlled Trial for Structural Heat Adaptation and Education in a rural setting of Pakistan

Authors

Das, Jai K.; Naqvi, Syeda Kanza; Bonell, Ana; burman, christopher; Augustin, Joseph; Qamar, Shahmeer; Rizvi, Arjumand; Chauhdary, Imran Ahmed; Naqvi, Anjum Abbas; Jamali, Muhammad Khan; Naqvi, Hassan; Tabassum, Farhana; Cousens, Simon; Bhutta, Zulfiqar A

VERSION 1 - REVIEW

**Reviewer**	**1**
**Name**	**Lu, Chan**
**Affiliation**	**Central South University, XiangYa School of Public Health**
**Date**	**23-May-2026**
**COI**	**None**

This manuscript presents a well-designed protocol for a cluster-randomized controlled trial evaluating a comprehensive heat adaptation bundle (ReHAB) in rural Pakistan. The topic is highly relevant given the escalating climate crisis and the vulnerability of low-resource rural populations. The intervention combines behavioral and structural components, and the planned use of wearable devices and environmental sensors is innovative. The protocol is generally clear and follows standard reporting guidelines. However, several methodological details require clarification or revision to enhance transparency, feasibility, and alignment with CONSORT guidelines for protocols.

Given the importance of this research and the potential for improvement, I suggest the editor accept this manuscript with major revisions.

I have some comments and suggestions for this manuscript as follows:

1. The protocol states that the pilot study will be conducted in a single village involving 20 households. It is unclear whether this pilot has already been completed or is planned. The manuscript would benefit from specifying the current status of the pilot, its expected

completion date, and how its findings will be used to refine the intervention before the main trial. Additionally, the dates for the main trial recruitment and follow-up should be explicitly stated (as required by BMJ Open for protocol papers).

2. The sample size section contains several points that need clarification. (a) The calculation for heat-related illness assumes a prevalence of 28.9% from a slum study in Ahmedabad, India. Please justify the relevance of this prevalence estimate to rural Pakistan, and discuss whether any local data were considered. (b) For the quality of life outcome, the mean baseline WHOQOL-BREF score of 68 (SD 18) is taken from Lodhi et al. 2019, which was a general Pakistani population study, not specifically a rural heat-exposed population. Please clarify whether this is appropriate. (c) The ICC values used (0.0122 for heat illness, 0.015 for QoL, 0.1736 for heart rate) are cited without references or justification. I suggest the authors should provide the sources or empirical basis for these ICC estimates, or explain that they are conservative assumptions based on similar cluster trials.

3. The control arm receives "routine care and existing government-led information." However, the intervention arm includes community group meetings, WhatsApp video messages, and door-to-door outreach. Given that clusters are villages, there is a potential risk of contamination if villages are geographically close and residents interact. I suggest the authors should describe the geographical separation between intervention and control clusters and any strategies (e.g., buffer zones) to minimize contamination. Additionally, the use of WhatsApp messages risks contamination if control arm residents are part of the same social networks; please discuss this limitation.

4. The primary outcome "heat-related illness" is defined using ICD-11 and institutional classification, but the protocol states that data will be collected through "weekly community survey, health facility data, community surveillance." The case definition for heat-related illness at the community level (without clinical assessment) is not clearly operationalized. I suggest the authors should provide the specific symptom-based criteria that community health workers or surveyors will use to identify a heat-related illness episode in the absence of a formal medical diagnosis. This is critical for reproducibility.

5. The protocol states that 5 mL venous blood samples will be collected at endline "for future analysis of heat-related biomarkers." However, it is not specified which specific biomarkers will be analyzed, nor whether there are any hypotheses or assays planned. I suggest the authors should clarify whether these samples are for exploratory biobanking only, or if there are specific pre-specified analyses (e.g., heat shock proteins, inflammatory markers). If the latter, these should be added as secondary or exploratory outcomes with appropriate ethical justification.

6. Some recent study found significant associations of increased temperature or extreme heat with allergy, infectious diseases and metabolic diseases (e.g., diabetes) (https://doi.org/10.1016/j.envint.2022.107456;
https://doi.org/10.1016/j.buildenv.2024.112317;

https://doi.org/10.1016/j.envint.2022.107456;
https://doi.org/10.1016/j.buildenv.2023.110668;
https://doi.org/10.1016/j.buildenv.2023.110800). Furthermore, some other new studies explain the potential mechanisms underlying the associations, such as the expressions of transient receptor potential (TRP) ion channels (TRPV1, TRPV4, TRPA1, TRPM2) (https://doi.org/10.1016/j.jhazmat.2026.142315; https://doi.org/10.1016/j.envpol.2025.126321; https://doi.org/10.1111/all.15883). I suggest the authors to discuss your findings with (some of) the new articles and to further support your findings and discuss the potential mechanism to support your results.

Reviewer 2

Name Lokmic-Tomkins, Zerina

Affiliation Monash University Faculty of Medicine Nursing and Health Sciences, School of Nursing and Midwifery

Date 27-May-2026

COI None

Apologies, I tried to attach this as a file but it would not upload. So copy/paste below:

Thank you for the opportunity to review this manuscript.

I recognise the importance and potential value of this work. However, I found aspects of the protocol difficult to follow, particularly given the complexity of the proposed RCT and the number of intervention components involved. As I read the manuscript, a number of questions arose from many angles regarding how the intervention will be operationalised and evaluated. I have attempted to organise these comments below in the hope that they may help strengthen and clarify the protocol for readers. I also apologise in advance for the number of questions raised; they are offered in a constructive spirit and with the intention of helping refine what could be a great, impactful study.

1. I found it difficult to determine how the intervention will be sufficiently standardised to ensure consistent delivery within the context of an RCT. The ReHAB intervention (which, if I may gently note, may benefit from reconsideration of the acronym) appears to include a broad bundle of components, including education, community mobilisation, structural household modifications, shading, ventilation, energy installations, hydration measures, and communal shelters. However, it is not entirely clear what each intervention household or cluster will specifically receive, or how consistency of delivery across sites will be maintained.

2. The protocol states that interventions will be selected based on factors such as building materials, structure, ventilation, shading, efficiency, and resident consent. This raises the possibility that the intervention could vary substantially between households and clusters. It

may therefore be helpful for the authors to clarify what constitutes the minimum intervention package, which components are mandatory versus optional, how household-level decisions regarding modifications will be made, and whether all clusters will receive a similar level of intervention intensity. In addition, I would appreciate further detail regarding how variation in structural modifications will be documented and whether the analysis will account for differential exposure to intervention components. Greater clarity in these areas would also improve the reproducibility of the intervention for future researchers or practitioners who may wish to adapt or implement this model in other settings globally.

3. I was also unclear whether the educational component is intended to function primarily as an educational intervention, a health-promotion intervention, or a combination of both. The manuscript describes awareness sessions, WhatsApp messages, demonstrations, and community group activities; however, if the intention is to deliver an educational and behaviour-change intervention, it may be useful for the protocol to provide more detail regarding the theoretical framework underpinning the educational component. For example, the protocol could clarify the key learning objectives, target behaviours, message content, frequency and duration of educational sessions, facilitator training, fidelity monitoring procedures, participant attendance tracking, and how knowledge, attitudes, and practices will be assessed. It would also be helpful to know whether any validated assessment tools will be used to evaluate behaviour change or educational outcomes.

4. I also wondered about the potential for contamination between study arms. If the education component includes WhatsApp messages, community meetings, and practical demonstrations, and given that neighbouring villages in rural settings often share information, labour, materials, and social networks, could this increase the risk of contamination between intervention and control clusters? It may therefore be helpful for the authors to clarify whether any measures will be taken regarding geographic separation between clusters, how contamination will be assessed, and whether sharing of WhatsApp messages or intervention-related information across arms will be monitored. If some degree of contamination is anticipated, it may also be worth considering whether this should be acknowledged as a study limitation and/or incorporated into sensitivity analyses.

5. The protocol appears to include at least two primary outcomes: heat-related illness and quality of life. However, it is not entirely clear which outcome constitutes the primary endpoint for determining trial success. I was uncertain whether both outcomes are expected to demonstrate benefit, or whether one is considered primary and the other secondary. Furthermore, heat-related illness is a complex outcome and may be difficult to measure reliably in a community-based trial. It would therefore be helpful for the authors to clarify whether heat-related illness will be self-reported, clinically confirmed, or surveillance-based. In addition, who will classify or diagnose heat-related illness events? How will mild heat-related symptoms be distinguished from clinically significant or diagnosable heat illness? Further clarification is also needed regarding how duplicate reports across household surveys, community logs, and facility records will be managed. It would also be helpful to

specify whether mortality is considered part of the primary outcome or a secondary outcome. I also wondered whether the involvement of community groups in surveillance activities within intervention clusters could introduce a risk of outcome misclassification or detection bias.

6. If I have understood the protocol correctly, the intervention arm will receive community mobilisation, education, and active surveillance, while control clusters will receive routine care. I wondered whether this could increase detection and reporting of heat-related illness in intervention clusters simply because participants may become more sensitised to recognising heat-related symptoms. It would therefore be helpful for the authors to explain how differential reporting between arms will be minimised. Ideally, outcome surveillance would be comparable across intervention and control clusters and, where feasible, conducted by independent assessors blinded to allocation. However, the current protocol does not make this sufficiently clear. I also found the description of the randomisation procedures somewhat difficult to follow. For example, in one section the manuscript states that 22 clusters will be randomised, whereas the line-listing section states that 60 clusters will be selected for randomisation. In the introduction (pages 4–5), the manuscript further states that: 'One village will serve as one cluster and 70–80 households from the village will be selected, and these clusters will be randomly assigned to either the intervention or control arm in a 1:1 ratio.' This made the rest of the protocol confusing and I would encourage the authors to clarify: how many clusters will initially be identified, how many clusters will ultimately be randomised, whether 60 clusters are screened and 22 selected, the criteria used to select the final clusters, whether selection occurs before or after baseline data collection and whether the selection process could introduce bias.

7. I would also strongly encourage the authors to develop a clear CONSORT- or PRISMA-style flow diagram illustrating the cluster selection and randomisation process -this will help also with the complexity of the RCT. While I appreciate the inclusion of multiple figures, I found several of them difficult to interpret. In particular, the dark background makes some diagrams challenging to read, and simpler figures with clearer stepwise information may improve readability substantially. Keep it simple.

8. I also struggled somewhat to understand the re-randomisation procedure. It would therefore be helpful for the authors to clarify the balance criteria being used, acceptable thresholds, the number of re-randomisations planned, and whether re-randomisation procedures will be fully prespecified prior to implementation.

9. The quality-of-life sample size calculation refers to 60 households per cluster, whereas elsewhere in the protocol the trial proposes enrolling 70–80 households per cluster. Additional clarification regarding the assumptions underlying the sample size calculation would therefore be very helpful. Specifically, the manuscript should clarify the assumed control event rate, the expected intervention effect in both absolute and relative terms, the number of individuals per cluster, and the source or assumed value for the intracluster

correlation coefficient (ICC) or coefficient of variation. It is also unclear whether the sample size calculation accounts for repeated measures over time or for household-level clustering within village clusters. Additional detail is needed regarding the assumptions used for the quality-of-life outcome and whether the study is adequately powered for both co-primary outcomes. It would also be helpful for the authors to clarify how multiplicity will be handled statistically, including whether any adjustment or correction procedures are planned. In addition, the manuscript would benefit from explicitly stating which reporting guidance will be used for the final RCT reporting. The protocol could align more clearly with SPIRIT and CONSORT extension guidance for cluster randomised trials and complex interventions – even though some sections in CONSORT list are N/A -these processes do require formulation at the protocol development stage- for example interim analysis process and stopping guidelines. Deviation from a protocol is a reportable ethics issue – defining all of this at the start avoids problems later. Inclusion of a SPIRIT checklist and trial schedule figure would also improve transparency.

10. I found the analysis plan difficult to follow and would strongly encourage review by a statistician and/or environmental epidemiologist to ensure sufficient clarity and methodological detail. From my understanding, it would be important for the protocol to clarify whether overdispersion will be assessed and how repeated weekly observations will be handled analytically. It would also be helpful to explain how seasonality and weather variation will be adjusted for, whether analyses will adjust for baseline outcome rates, and how missing data will be managed. Further clarification is also needed regarding whether intervention effects will be estimated at the individual, household, or cluster level. For the quality-of-life outcome specifically, the statement that mean increases will be compared may not be sufficient for a cluster RCT involving repeated measures, and a more detailed statistical approach may be warranted.

11. I also note that the protocol states that communities will contribute at least 30% of the intervention cost. This requires further ethical justification, particularly in relation to whether the poorest households, who may also be among those most affected by heat, could be excluded because they are unable to contribute. The authors should also clarify whether non-monetary contributions are genuinely feasible in these communities and whether contribution requirements could inadvertently worsen inequities. From an ethical perspective, several additional issues also require clarification: who will ultimately own the installed materials, what will happen if structural modifications fail or damage property, and whether control communities will receive any delayed benefit after the trial.

12. The protocol mentions endline blood sampling and future analysis of heat-related biomarkers, but this component is not well integrated into the trial aims. Further ethical and scientific justification is needed. The authors should explain which biomarkers are planned, why these biomarkers are relevant, and whether the biomarker analyses are exploratory. It would also be helpful to clarify why blood samples are necessary if they are not directly related to the RCT's primary outcomes. The protocol should describe how consent for future

use will be managed and whether the proposed degree of biobanking is proportionate to the study's primary aims.

13. The use of wearables and environmental sensors is a major strength of the protocol. However, additional operational detail is needed. The authors should clarify whether the devices have been validated in hot rural settings, how participant compliance will be monitored, what criteria will be used to define valid device wear time, how missing or implausible data will be handled, how environmental sensors will be calibrated, what rules will guide indoor device placement, and whether device placement will differ between intervention and control homes.

Minor comments:

The term "routine care and existing government-led information" needs to be more clearly defined. It would be helpful for the authors to specify what the control group will realistically be able to access during the study period, including government heat warnings, health worker advice, NGO interventions, disaster-response activities, and media campaigns. This is important because any external heat-adaptation activities available to control communities could dilute the estimated intervention effect.

Implementation fidelity requires substantially more detail. The protocol would benefit from clearer and more organised information regarding intervention delivery checklists, the number and duration of education sessions delivered, attendance records, WhatsApp message delivery and viewing metrics, household retrofit completion records, quality assurance procedures for construction work, monitoring of maintenance and continued use, and the dose of intervention received by each household.

Once again, my apologies for so many comments -community-based research is complex and ethically wrought. I am hopeful that they will be of help to you.

VERSION 1 - AUTHOR RESPONSE

S. No.	Source / Comment No.	Manuscript section	Reviewer Comment	Response / Action taken
11	Reviewer 1 – 1	Methods / Pilot study and timeline	The protocol states that the pilot study will be conducted in a single village involving 20 households. It is unclear whether this pilot has already been completed or is planned. The manuscript would benefit from specifying the current status of the pilot, its expected completion date, and how its findings will be used to refine the intervention before the main trial. Additionally, the dates for the main trial recruitment and follow-up should be explicitly stated (as required by BMJ Open for protocol papers).	**Response:** We thank the reviewer for this helpful suggestion. We have clarified the status of the pilot study, including its timing and objectives. We have also added the planned recruitment and follow-up dates for the main trial in accordance with BMJ Open protocol reporting requirements.

				Manuscript Changes: Under the heading "Pilot Study". Page 7.
12	Reviewer 1 – 2	Methods / Sample size	The sample size section contains several points that need clarification. (a) The calculation for heat-related illness assumes a prevalence of 28.9% from a slum study in Ahmedabad, India. Please justify the relevance of this prevalence estimate to rural Pakistan, and discuss whether any local data were considered. (b) For the quality of life outcome, the mean baseline WHOQOL-BREF score of 68 (SD 18) is taken from Lodhi et al. 2019, which was a general Pakistani population study, not specifically a rural heat-exposed population. Please clarify whether this is appropriate. (c) The ICC values used (0.0122 for heat illness, 0.015 for QoL, 0.1736 for heart rate) are cited without references or justification. I suggest the authors should provide the sources or empirical basis for these ICC estimates, or explain that they are conservative assumptions based on similar cluster trials.	**Response:** We thank the reviewer for this observation. Population-based estimates of heat-related illness prevalence from rural Pakistan were not available at the time of study design; therefore, we used the prevalence reported from a comparable heat-vulnerable South Asian setting in Ahmedabad, India. Similarly, no published WHOQOL-BREF data were available from rural heat-exposed populations in Pakistan, and we therefore used estimates from a large population-based Pakistani study to provide locally relevant parameters for sample size estimation (which is a conservative estimate). We have also clarified the basis of the ICC assumptions and provided additional justification in the manuscript and added references.<br><br>Manuscript Changes: Under the heading "Sample Size". Page 6.
13	Reviewer 1 – 3	Methods / Intervention, control arm, contamination	The control arm receives "routine care and existing government-led information." However, the intervention arm includes community group meetings, WhatsApp video messages, and door-to-door outreach. Given that clusters are villages, there is a potential risk of contamination if villages are geographically close and residents interact. I suggest the authors should describe the geographical separation	**Response:** We thank the reviewer for this important observation. Community meetings, door-to-door outreach, and WhatsApp messaging are already described as components of the intervention package in the manuscript. To further

			between intervention and control clusters and any strategies (e.g., buffer zones) to minimize contamination. Additionally, the use of WhatsApp messages risks contamination if control arm residents are part of the same social networks; please discuss this limitation.	clarify measures taken to minimize contamination, we have added information on distance in cluster selection (1-2km). In addition, WhatsApp messages were distributed only to intervention participants and shared using disappearing-message settings to reduce onward dissemination as mentioned in the protocol. Nevertheless, we acknowledge that some residual contamination through social interactions and social networks cannot be completely excluded and have noted this as a study limitation.<br><br>Manuscript Changes: Under the heading “Community education and Awareness”. Page 8-9. And Under “cluster randomization”. Page 11.
14	Reviewer 1 – 4	Methods / Outcomes and data collection	The primary outcome "heat-related illness" is defined using ICD-11 and institutional classification, but the protocol states that data will be collected through "weekly community survey, health facility data, community surveillance." The case definition for heat-related illness at the community level (without clinical assessment) is not clearly operationalized. I suggest the authors should provide the specific symptom-based criteria that community health workers or surveyors will use to identify a heat-related illness episode in the absence of a formal medical diagnosis. This is critical for reproducibility.	**Response:** We thank the reviewer for this important comment. The symptom-based criteria used to identify and classify heat-related illness episodes at the community level are provided in Figure 2. To improve clarity, we have added text explicitly referring readers to Figure 2 and clarifying that reports from community surveillance, household surveys, and health facility will be classified using this standardized algorithm and would be assessed by study data collectors who will use the same survey tool

				Manuscript Changes: under the heading "Household Data Collection". Page 13
15	Reviewer 1 – 5	Methods / Biomarkers and ethics	The protocol states that 5 mL venous blood samples will be collected at endline "for future analysis of heat-related biomarkers." However, it is not specified which specific biomarkers will be analyzed, nor whether there are any hypotheses or assays planned. I suggest the authors should clarify whether these samples are for exploratory biobanking only, or if there are specific pre-specified analyses (e.g., heat shock proteins, inflammatory markers). If the latter, these should be added as secondary or exploratory outcomes with appropriate ethical justification.	**Response:** We thank the reviewer for this comment. As described in the manuscript, the blood samples are being collected for biobanking purposes only and are not linked to any prespecified biomarker analyses within the current trial (Ethical considerations, page 18). Accordingly, no biomarker outcomes, hypotheses, or assays form part of the trial objectives or outcome framework.<br><br>Manuscript Changes: we have added that these would be exploratory and have not been specified yet. Page xx
16	Reviewer 1 – 6	Background / Discussion	Some recent study found significant associations of increased temperature or extreme heat with allergy, infectious diseases and metabolic diseases (e.g., diabetes) (https://doi.org/10.1016/j.envint.2022.107456; https://doi.org/10.1016/j.buildenv.2024.112317; https://doi.org/10.1016/j.envint.2022.107456; https://doi.org/10.1016/j.buildenv.2023.110668; https://doi.org/10.1016/j.buildenv.2023.110800). Furthermore, some other new studies explain the potential mechanisms underlying the associations, such as the expressions of transient receptor potential (TRP) ion channels (TRPV1, TRPV4, TRPA1, TRPM2) (https://doi.org/10.1016/j.jhazmat.2026.142315; https://doi.org/10.1016/j.envpol.2025.126321;	**Response:** We thank the reviewer for highlighting these studies. While we acknowledge the broader evidence on the health impacts of heat exposure, a detailed discussion of additional outcomes and specific biological mechanisms are beyond the scope of the current protocol.<br><br>Manuscript Changes: None

			https://doi.org/10.1111/all.15883). I suggest the authors to discuss your findings with (some of) the new articles and to further support your findings and discuss the potential mechanism to support your results.	
17	Reviewer 2 – 1	Methods / Intervention standardisatio n	I found it difficult to determine how the intervention will be sufficiently standardised to ensure consistent delivery within the context of an RCT. The ReHAB intervention (which, if I may gently note, may benefit from reconsideration n of the acronym) appears to include a broad bundle of components, including education, community mobilisation, structural household modifications, shading, ventilation, energy installations, hydration measures, and communal shelters. However, it is not entirely clear what each intervention household or cluster will specifically receive, or how consistency of delivery across sites will be maintained.	**Response:** We thank the reviewer for this comment. As this is a cluster-randomized trial, intervention delivery is standardized at the cluster level. All intervention clusters receive the same education and community mobilization package, while structural interventions are delivered proportionately across clusters according to predefined criteria. We have clarified this in the manuscript.<br><br>Manuscript Changes: Under the heading “structural modification”. Page 10.
18	Reviewer 2 – 2	Methods / Intervention components and fidelity	The protocol states that interventions will be selected based on factors such as building materials, structure, ventilation, shading, efficiency, and resident consent. This raises the possibility that the intervention could vary substantially between households and clusters. It may therefore be helpful for the authors to clarify what constitutes the minimum intervention package, which components are mandatory versus optional, how household-level decisions regarding modifications will be made, and whether all clusters will receive a similar level of intervention intensity. In addition, I would appreciate further detail regarding how variation in structural modifications will be documented and whether the analysis will account for differential exposure to intervention components. Greater clarity in these areas would also improve the	**Response:** We thank the reviewer for this comment. As this is a cluster-randomized trial, intervention delivery is standardized at the cluster level rather than the household level, which means that each cluster in the intervention cluster will have equal number of structures intervention both overall and category. We have clarified the intervention standardization approach and provided additional detail on implementation and documentation procedures. This is also clarified in the analysis

			reproducibility of the intervention for future researchers or practitioners who may wish to adapt or implement this model in other settings globally.	section and the planned sub-group analysis by type of intervention,<br><br>Manuscript Changes: Under the heading “Intervention”. Page 8-10.<br><br>Also Analysis. Page xx
19	Reviewer 2 – 3	Methods / Educational and behavioural intervention	I was also unclear whether the educational component is intended to function primarily as an educational intervention, a health-promotion intervention, or a combination of both. The manuscript describes awareness sessions, WhatsApp messages, demonstrations, and community group activities; however, if the intention is to deliver an educational and behaviour-change intervention, it may be useful for the protocol to provide more detail regarding the theoretical framework underpinning the educational component. For example, the protocol could clarify the key learning objectives, target behaviours, message content, frequency and duration of educational sessions, facilitator training, fidelity monitoring procedures, participant attendance tracking, and how knowledge, attitudes, and practices will be assessed. It would also be helpful to know whether any validated assessment tools will be used to evaluate behaviour change or educational outcomes.	**Response:** Much of the requested information, including message content, delivery methods, frequency of sessions, facilitator training, and behavior assessment, is described in the intervention section which has been now enhanced for clarity. The educational component was developed through the formative phase and community consultations rather than a specific behavioral theory framework. We have clarified this in the manuscript where appropriate.<br><br>Manuscript Changes: Under the heading “Community education and Awareness”. Page 8-9.
20	Reviewer 2 – 4	Methods / Contamination	I also wondered about the potential for contamination between study arms. If the education component includes WhatsApp messages, community meetings, and practical demonstrations, and given that neighbouring villages in rural settings often share information, labour, materials, and social networks, could this increase the risk of contamination between intervention and control clusters? It may therefore be helpful for the authors to clarify whether any measures will be taken regarding geographic separation between clusters, how contamination	**Response:** We have clarified that non-adjacent villages will be selected, with a minimum separation of approximately 1–2 Km. WhatsApp messages will be shared using disappearing-message settings. We acknowledge that some residual contamination through social networks cannot be fully excluded

			will be assessed, and whether sharing of WhatsApp messages or intervention-related information across arms will be monitored. If some degree of contamination is anticipated, it may also be worth considering whether this should be acknowledged as a study limitation and/or incorporated into sensitivity analyses.	and have noted this as a limitation.<br><br>Manuscript Changes: Under the heading "cluster randomization" page 11-12 and Under the heading ""Community education and Awareness" page 8-9.
21	Reviewer 2 – 5	Methods / Outcomes and surveillance	The protocol appears to include at least two primary outcomes: heat-related illness and quality of life. However, it is not entirely clear which outcome constitutes the primary endpoint for determining trial success. I was uncertain whether both outcomes are expected to demonstrate benefit, or whether one is considered primary and the other secondary. Furthermore, heat-related illness is a complex outcome and may be difficult to measure reliably in a community-based trial. It would therefore be helpful for the authors to clarify whether heat-related illness will be self-reported, clinically confirmed, or surveillance-based. In addition, who will classify or diagnose heat-related illness events? How will mild heat-related symptoms be distinguished from clinically significant or diagnosable heat illness? Further clarification is also needed regarding how duplicate reports across household surveys, community logs, and facility records will be managed. It would also be helpful to specify whether mortality is considered part of the primary outcome or a secondary outcome. I also wondered whether the involvement of community groups in surveillance activities within intervention clusters could introduce a risk of outcome misclassification or detection bias.	Response: These are very useful points that are needed for clearer description and thank you for highlighting these. We have clarified the primary and secondary outcomes, heat-illness ascertainment procedures, and management of duplicate reports across data sources.<br>Heat-related illness and quality of life are both prespecified co-primary outcomes, as described in the objectives and outcomes sections and the final analysis will help determine that on which outcomes the intervention had an effect – whether its one or both primary outcomes and the findings would be assessed accordingly. We understand the complexity of measuring heat illness in the community and most of these are managed at home, Hence we have tried the best to collect, categorize and standardize the process, through standardized symptom check list, categorization through ICD collection by trained data collectors. All the

				other sources of data which are from community and health facility would be revisited by the data collection team to ensure homogeneity. We have added clarifying text in the manuscript to avoid any ambiguity.<br><br>Manuscript Changes: under the heading "Statistical Analysis" page 16-17.
22	Reviewer 2 – 6	Methods / Surveillance, randomisation and selection of clusters	If I have understood the protocol correctly, the intervention arm will receive community mobilisation, education, and active surveillance, while control clusters will receive routine care. I wondered whether this could increase detection and reporting of heat-related illness in intervention clusters simply because participants may become more sensitised to recognising heat-related symptoms. It would therefore be helpful for the authors to explain how differential reporting between arms will be minimised. Ideally, outcome surveillance would be comparable across intervention and control clusters and, where feasible, conducted by independent assessors blinded to allocation. However, the current protocol does not make this sufficiently clear. I also found the description of the randomisation procedures somewhat difficult to follow. For example, in one section the manuscript states that 22 clusters will be randomised, whereas the line-listing section states that 60 clusters will be selected for randomisation. In the introduction (pages 4–5), the manuscript further states that: 'One village will serve as one cluster and 70–80 households from the village will be selected, and these clusters will be randomly assigned to either the intervention or control arm in a 1:1 ratio.' This made the rest of the protocol confusing and I would encourage the authors to clarify: how many clusters will initially be identified, how many	Response: The outcome ascertainment will be conducted in both intervention and control clusters using the same data collection procedures and outcome definitions. We have now clarified this in the manuscript.<br><br>Furthermore, we have clarified on the cluster selection and randomization process, including the relationship between the 85 villages screened, the 60 villages shortlisted following feasibility assessment, and the final 22 clusters randomized to the trial.<br><br>Manuscript Changes: Under the heading "Household data collection" page 13. And under "line listing" page 11.

			clusters will ultimately be randomised, whether 60 clusters are screened and 22 selected, the criteria used to select the final clusters, whether selection occurs before or after baseline data collection and whether the selection process could introduce bias.	
23	Reviewer 2 – 7	Figures / Trial flow diagram	I would also strongly encourage the authors to develop a clear CONSORT- or PRISMA-style flow diagram illustrating the cluster selection and randomisation process -this will help also with the complexity of the RCT. While I appreciate the inclusion of multiple figures, I found several of them difficult to interpret. In particular, the dark background makes some diagrams challenging to read, and simpler figures with clearer stepwise information may improve readability substantially. Keep it simple.	Response: We thank the reviewer for this comment. The submitted figures were prepared with transparent backgrounds; however, these may have appeared differently within the review system. We have reviewed the figures and will ensure that the final submitted versions are provided in a format that preserves readability and clarity.<br>We have also revised the study flow diagram as suggested.<br><br>Manuscript Changes: Figures uploaded separately.
24	Reviewer 2 – 8	Methods / Re-randomisation	I also struggled somewhat to understand the re-randomisation procedure. It would therefore be helpful for the authors to clarify the balance criteria being used, acceptable thresholds, the number of re-randomisations planned, and whether re-randomisation procedures will be fully prespecified prior to implementation.	Response: We have clarified the constrained re-randomization procedure, including the prespecified balance variables, use of multiple allocation schemes, independent statistician involvement, and assessment of balance before final allocation.<br><br>Manuscript Changes: Under the heading “Cluster Randomization” Page 11.
25	Reviewer 2 – 9	Methods / Sample size and reporting guidelines	The quality-of-life sample size calculation refers to 60 households per cluster, whereas elsewhere in the protocol the trial proposes enrolling 70–80 households per cluster. Additional clarification regarding the assumptions underlying the sample size calculation	Response: We thank the reviewer for these helpful comments. The sample size section already includes the main assumptions for both co-primary

			would therefore be very helpful. Specifically, the manuscript should clarify the assumed control event rate, the expected intervention effect in both absolute and relative terms, the number of individuals per cluster, and the source or assumed value for the intracluster correlation coefficient (ICC) or coefficient of variation. It is also unclear whether the sample size calculation accounts for repeated measures over time or for household-level clustering within village clusters. Additional detail is needed regarding the assumptions used for the quality-of-life outcome and whether the study is adequately powered for both co-primary outcomes. It would also be helpful for the authors to clarify how multiplicity will be handled statistically, including whether any adjustment or correction procedures are planned. In addition, the manuscript would benefit from explicitly stating which reporting guidance will be used for the final RCT reporting. The protocol could align more clearly with SPIRIT and CONSORT extension guidance for cluster randomised trials and complex interventions – even though some sections in CONSORT list are N/A -these processes do require formulation at the protocol development stage- for example interim analysis process and stopping guidelines. Deviation from a protocol is a reportable ethics issue – defining all of this at the start avoids problems later. Inclusion of a SPIRIT checklist and trial schedule figure would also improve transparency.	outcomes, including prevalence/mean estimates, effect sizes, ICCs, power, alpha, attrition, and number of clusters. We have clarified that the final enrolment target was based on the larger sample size required for the heat-primary illness outcome, while the quality-of-life outcome required 60 households per cluster. We have also refined the quality-of-life analysis plan to clarify repeated assessments over time and added a statement regarding the analysis of the two co-primary outcomes. A completed SPIRIT checklist has been provided as supplementary material, and the assessment schedule (table 3) has been revised for clarity.<br><br>Manuscript Changes: Under the heading “sample size”, “statistical analysis”, and “Table 3” at page 6-7, 16-17, and 15 respectively.
26	Reviewer 2 – 10	Methods / Statistical analysis	I found the analysis plan difficult to follow and would strongly encourage review by a statistician and/or environmental epidemiologist to ensure sufficient clarity and methodological detail. From my understanding, it would be important for the protocol to clarify whether overdispersion will be assessed and how repeated weekly observations will be handled analytically. It would also be helpful to explain how seasonality and weather variation will be adjusted for, whether analyses will adjust for baseline outcome rates, and	Response: We thank the reviewer for this helpful comment. We have revised the statistical analysis section to improve clarity and methodological detail, including the unit of inference, handling of clustering and repeated measures, adjustment for seasonality and baseline covariates, assessment of

			how missing data will be managed. Further clarification is also needed regarding whether intervention effects will be estimated at the individual, household, or cluster level. For the quality-of-life outcome specifically, the statement that mean increases will be compared may not be sufficient for a cluster RCT involving repeated measures, and a more detailed statistical approach may be warranted.	overdispersion, management of missing data, and the planned mixed-effects approach for the quality-of-life outcome.<br><br>Manuscript Changes: under the heading "statistical analysis" page 16-17.
27	Reviewer 2 – 11	Methods / Ethics and equity	I also note that the protocol states that communities will contribute at least 30% of the intervention cost. This requires further ethical justification, particularly in relation to whether the poorest households, who may also be among those most affected by heat, could be excluded because they are unable to contribute. The authors should also clarify whether non-monetary contributions are genuinely feasible in these communities and whether contribution requirements could inadvertently worsen inequities. From an ethical perspective, several additional issues also require clarification: who will ultimately own the installed materials, what will happen if structural modifications fail or damage property, and whether control communities will receive any delayed benefit after the trial.	Reference: We have clarified that community contribution is considered at the cluster level and may be monetary or non-monetary, and that individual households will not be excluded solely because of inability to contribute financially. We have also clarified ownership of installed household and communal materials. Structural safety procedures are described in the manuscript and further addressed in the consent form.<br>No delayed benefits have been planned at this stage for control clusters.<br><br>Manuscript Changes: Under the heading "structural modifications" and "ethical consideration and dissemination". Page 9-10, and 18-19 respectively.
28	Reviewer 2 – 12	Methods / Biomarkers, biobanking and consent	The protocol mentions endline blood sampling and future analysis of heat-related biomarkers, but this component is not well integrated into the trial aims. Further ethical and scientific justification is needed. The authors should explain which biomarkers are planned, why these biomarkers are relevant, and whether the biomarker	Response: We thank the reviewer for this comment. The blood samples are being collected for biobanking purposes only and are not linked to any prespecified biomarker analyses or trial

			analyses are exploratory. It would also be helpful to clarify why blood samples are necessary if they are not directly related to the RCT's primary outcomes. The protocol should describe how consent for future use will be managed and whether the proposed degree of biobanking is proportionate to the study's primary aims.	outcomes. As already described in the manuscript, separate consent will be obtained for blood sample collection, storage, and future ethically approved use, with provisions for de-identification and withdrawal of consent.<br><br>Manuscript Changes: None
29	Reviewer 2 – 13	Methods / Wearables and environmental sensors	The use of wearables and environmental sensors is a major strength of the protocol. However, additional operational detail is needed. The authors should clarify whether the devices have been validated in hot rural settings, how participant compliance will be monitored, what criteria will be used to define valid device wear time, how missing or implausible data will be handled, how environmental sensors will be calibrated, what rules will guide indoor device placement, and whether device placement will differ between intervention and control homes.	Response: We thank the reviewer for this comment. The devices used within the study are globally validated and used as standard for environmental monitoring. We have clarified the operational details in the protocol including compliance checks, missing data etc.<br><br>Manuscript Changes: Table 2 page 14 and under the heading "Data collection for subset population" page 15.
30	Reviewer 2 – 14	Methods / Control arm	The term "routine care and existing government-led information" needs to be more clearly defined. It would be helpful for the authors to specify what the control group will realistically be able to access during the study period, including government heat warnings, health worker advice, NGO interventions, disaster-response activities, and media campaigns. This is important because any external heat-adaptation activities available to control communities could dilute the estimated intervention effect.	Response: We have clarified what routine care includes and specified that control clusters will not receive ReHAB-specific intervention components.<br><br>Manuscript Changes: under the heading "control arm" page 11.
31	Reviewer 2 – 15	Methods / Implementation fidelity	Implementation fidelity requires substantially more detail. The protocol would benefit from clearer and more organised information regarding intervention delivery checklists, the number and duration of education sessions delivered, attendance records, WhatsApp message delivery and	Response: we have now further elaborated on the fidelity-related procedures in the manuscript. We have further clarified that implementation fidelity will be monitored

			viewing metrics, household retrofit completion records, quality assurance procedures for construction work, monitoring of maintenance and continued use, and the dose of intervention received by each household.	through session records, CG logbooks, WhatsApp delivery records, retrofit completion records, and supervision by research and technical teams.<br><br>Manuscript Changes: under the heading "community education and awareness" page 8

VERSION 2 - REVIEW

**Reviewer**	**1**
**Name**	**Lu, Chan**
**Affiliation**	**Central South University, XiangYa School of Public Health**
**Date**	**08-Jul-2026**
**COI**	

Many thanks for the authors' careful explanations and revision. The authors have addressed all my comments. Now, I have no further comments.
