# Supplementary material for: Structural heat adaptation and education in a rural setting of Pakistan: protocol for a randomised controlled trial
Source: BMJ Open. 2026 Aug 31;16(8):e121448. doi: 10.1136/bmjopen-2026-121448 (PMC13536121; doi:10.1136/bmjopen-2026-121448)
Supplement: Supplementary file 2 [file bmjopen-16-8-s001.docx]

**Additional Informed Consent Form for Blood Sample Collection and Biobanking**

| **Title of Research Project** | Structural Heat Adaptation, Education and Prevention in Urban and Rural Settings |
| --- | --- |
| **Principal Investigator** | Dr. Zulfiqar A Bhutta Phone: 92 0213 486 9825 |
| **Institute** | IGHD - Aga Khan University |

**Introduction:**

I am____________________________from the Institute for Global Health Development, Aga Khan University and we are conducting a research study on Structural Heat Adaptation, Education and Prevention in Urban and Rural Settings.

You have been selected to participate in this research study. This additional consent form explains the optional collection and storage of a blood sample for future ethically approved research. We will provide you with a briefing about this procedure, and written consent will be obtained from you before any blood sample is collected. Refusing to provide a blood sample will not affect your participation in the main study or any services available to you.

**Purpose of this research procedure:**

The purpose of collecting a blood sample is to store it securely in a biobank for possible future analysis of heat-related biomarkers. These future analyses may help researchers better understand the biological effects of heat exposure and heat adaptation. Any future use of the samples will require appropriate ethical approval.

**Procedures**

We seek your permission for the following:

1. Collection of approximately 5 mL of venous blood at endline during the peak heat period in the second year of the study.
2. The blood sample will be collected by a trained phlebotomist using sterile techniques and standard precautions.
3. The sample will be processed and stored for at least seven years in a secure, access-controlled biobank according to established protocols and national standards.
4. The sample will be labelled using a unique study code. Your name and other personal identifiers will be stored separately in password-protected records accessible only to authorised study personnel.
5. The sample may be used in future ethically approved research related to heat exposure, heat adaptation, and heat-related biomarkers.

**Possible risks or benefits:**

**Individual participant risks:**

a) Blood collection may cause mild pain or a pinch at the needle site.

b) There may be minor bruising, bleeding, swelling, or dizziness after the sample is collected.

c) There is a very small risk of infection, which will be minimised by using sterile equipment and trained staff.

**Benefits:**

There may be no direct medical benefit to you from providing the blood sample.

***Indirect Benefits:*** Your participation may contribute to future research that improves understanding of heat-related health effects and supports the development of effective heat adaptation strategies for vulnerable populations.

**Right of refusal to participate and withdrawal**

Your participation in blood sample collection is voluntary. You may refuse to provide a blood sample and still continue in the main study. You may also withdraw your consent for storage of your sample at any time. If you withdraw, you may request destruction of your remaining stored sample, unless it has already been used in an approved analysis.

**Confidentiality**

Your sample and related information will remain confidential. The sample will be de-identified using a unique study code, and personal identifiers will be stored separately in password-protected databases accessible only to authorised personnel. Ethics review committees or regulatory authorities may inspect study records. Findings from future analyses may be published in journals or reports, but your name and identity will not be disclosed.

**Available sources of information**

If you have any questions about this procedure or the study, you may ask me now or contact the study principal investigator, Dr. Zulfiqar A Bhutta, Director, Institute for Global Health and Development, The Aga Khan University, Stadium Road, Karachi, on phone # 0213-4869825.

**Authorization**

I have read and understand this additional consent form. I voluntarily agree to provide a 5 mL blood sample for storage in a secure biobank and for possible future ethically approved research related to heat exposure, heat adaptation, and heat-related biomarkers. I understand that I may withdraw my consent for storage and request destruction of my remaining stored sample at any time. I understand that I will receive a copy of this form and that my consent does not take away any legal rights in the case of negligence or other legal fault of anyone involved in this study.

| Name of Participant: | ____________________________________ |
| --- | --- |
| Signature or thumb impression of Participant: | ____________________________________ |
| Date: | ____________________________________ |
| ***For Participants unable to read*** |  |
| **Witness: I have witnessed the accurate reading of the consent form to the potential participant, and the individual has had the opportunity to ask questions. I confirm that the individual has given consent freely.** |  |
| Name of witness: | ____________________________________ |
| Signature of witness: | ____________________________________ |
| Date: | ____________________________________ |
| Name of the person obtaining consent | ____________________________________ |
| Signature of the person obtaining consent | ____________________________________ |
| Date: | ____________________________________ |
| Name of Principal Investigator: | Dr. Zulfiqar A Bhutta (Aga Khan University) |
| Signature of Principal Investigator: | ____________________________________ |
| Date: | ____________________________________ |
