# Supplementary material for: Structural heat adaptation and education in a rural setting of Pakistan: protocol for a randomised controlled trial
Source: BMJ Open. 2026 Aug 31;16(8):e121448. doi: 10.1136/bmjopen-2026-121448 (PMC13536121; doi:10.1136/bmjopen-2026-121448)
Supplement: Supplementary file 3 [file bmjopen-16-8-s002.docx]

**Informed Consent Form**

| **Title of Research Project** | Structural Heat Adaptation, Education and Prevention in Urban Settings |
| --- | --- |
| **Principal Investigator** | Dr. Zulfiqar A Bhutta  Phone: 92 0213 486 9825 |
| **Institute** | IGHD - Aga Khan University |

**Introduction:**

I am____________________________from the Institute for Global Health Development, Aga Khan University and we are doing a research study on Structural Heat Adaptation, Education and Prevention in Urban Settings.

The study will be conducted in Karachi, Sindh. Since you belong to the targeted area and have been selected based on the criteria, I would like to invite you to participate in this research study. We will provide you with a briefing about the study, and written consent will be obtained from you. Once you provide your consent, we will include you in our study and conduct detailed interviews and assessments regarding you, your family, your house structure, and the lifestyle of you and your family. Assessment and interview can take your 30 minutes time.

#### Purpose of this research study:

The purpose of the study is to develop cost-effective interventions for heat adaptation through community engagement and evaluate the effectiveness of those interventions for vulnerable populations in urban and rural areas of Pakistan.

#### Procedures

As your household has been selected to participate in our research, we seek your permission for the following:

1. Receiving specific heat adaptation interventions, which may include educational sessions, structural modifications to your home, and personal cooling strategies.
2. You may be asked to wear a device that monitors your heart rate and activity levels during the hottest months of the year.
3. Health data, including any heat-related illnesses or hospital visits, will be collected and analyzed.
4. Regular follow-ups will be conducted to assess the effectiveness of the interventions over the course of the study.
5. Collection of 5ml blood sample for storage and be used later on for any other biological purposes.

#### Possible risks or benefits:

#### *Individual participant risks:*

a) Wearing a monitoring device might cause minor discomfort or inconvenience.
b) Participating in multiple surveys and interviews may be time-consuming and cause fatigue.
c) Collecting the blood sample may cause a pinch.

***Structural Modification Risks:***

1) There is a risk of injury during the construction or modification process, such as cuts, falls, or other accidents. All construction activities will be carried out by trained professionals to minimize these risks.
2) There is a risk that modifications might affect the structural integrity of your home. Assessments will be conducted prior to any modifications to ensure they do not compromise safety.
3) The construction process may cause noise and other disturbances, which could be disruptive to your routine and comfort.

#### *Benefits:*

The potential benefits of participating in this study include:

***Direct Benefits:*** You may experience improved heat management and reduced health risks from heat through the interventions provided.

***Indirect Benefits:*** Your participation will contribute to valuable research that could help develop effective heat adaptation strategies for vulnerable populations in similar settings. The findings of this study may lead to improved public health policies and practices in your community and beyond.

**Right of refusal to participate and withdrawal**

#### You are free to choose to participate in the study. You may refuse to participate as your participation is voluntary. You can also refuse to answer some questions provided in the survey form or refuse to have us take photographs of your home if you do not feel comfortable. In case you decide to withdraw your participation in the study, we will not process the information collected from you, and will not go for any proposed structural modification of your house, such information will be deleted from our records.

#### Confidentiality

The information provided by you will remain confidential. Nobody except the study team members will have access to it. Your name and identity will also not be disclosed at any time. However, the data may be inspected by Ethics Review Committee at Aga khan university and may be published in journals and elsewhere without giving your name or disclosing your identity.

**Available sources of information**

If you have any questions about this study, you may ask me now or can contact the study principal investigator Dr Zulfqar A Bhutta Director Institute for Global Health and Development, The Aga Khan University, Stadium Road, Karachi on phone # 0213-4869825.

**Authorization**

I have read and understand this consent form, and I volunteer to participate in this research study. I understand that I will receive a copy of this form. I voluntarily choose to participate, but I understand that my consent does not take away any legal rights in the case of negligence or other legal fault of anyone who is involved in this study. I further understand that nothing in this consent form is intended to replace any applicable Federal, state, or local laws.

| ____________________________________________ | Name of Participant: |
| --- | --- |
| ____________________________________________ | Signature or thumb impression of Participant: |
| ____________________________________________ | Date: |
|  | ***For Participants unable to read*** |
| **Witness:**  I have witnessed the accurate reading of the consent form to the potential participant, and the individual has had the opportunity to ask questions. I confirm that the individual has given consent freely. | |
| ____________________________________________ | Name of witness: |
| ____________________________________________ | Signature of witness: |
| ____________________________________________ | Date: |
|  |  |
| ____________________________________________ | Name of the person obtaining consent |
| ____________________________________________ | Signature of the person obtaining consent |
| ____________________________________________ | Date: |
|  |  |
| Dr. Zulfiqar A Bhutta (Aga Khan University) | Name of Principal Investigator: |
| ____________________________________________ | Signature of Principal Investigator: |
| ____________________________________________ | Date: |
